# Phenological mismatch between alpine flowers and bumble bees: its mechanism and impacts on the population dynamics of bumble bees

**DOI:** 10.1007/s00442-025-05775-4

**Published:** 2025-08-23

**Authors:** Gaku Kudo, Tetsuo Imoto, Taietsu Nagase, Hai Xiang Liew

**Affiliations:** 1 Faculty of Environmental Earth Science, Hokkaido University, Sapporo, 060-0810 Japan; 2 Bumblebee Research Network, Suehiro Higashi 2-4, Asahikawa, 071-8122 Japan; 3 Graduate School of Environmental Science, Hokkaido University, Sapporo, 060-0810 Japan

**Keywords:** Alpine ecosystem, Bumble bee, Flowering phenology, Global warming, Phenological mismatch

## Abstract

To determine the impacts of global warming on pollinator-plant interactions, we recorded phenological variations in alpine flowers and bumble bees during 10–12 years in northern Japan, and analyzed the effects of weather conditions and phenological shift on worker population dynamics of four *Bombus* species. Flowering patterns of alpine plants were formed by the combination of early-flowering fellfield and late-flowering snowbed communities, where snowbed flowers were important resources for worker bees. The flowering phenology of the fellfield communities was correlated with early season air temperature, whereas that of the snowbed communities was clearly predicted by snowmelt time. It was predicted that 1 °C warming with 10 days earlier snowmelt would advance the peak flowering time of the fellfield and snowbed communities by 3.6 and 9.5 days, respectively, resulting in a 9.2 day shorter flowering period. In contrast, the peak time of worker abundance was consistent between years, independent of temperature and snowmelt time. As a result, the time lag between the peak flowering of snowbed plants and the peak abundance of worker bees, i.e., the phenological mismatch, increased with earlier snowmelt. Mid-summer temperature was negatively correlated with worker abundance for three *Bombus* species, and the effect of phenological mismatch varied between *Bombus* species. The abundance of *B. hypocrita* decreased with increasing phenological mismatch in the previous year, the abundance of *B. beaticola* and *B. yezoensis* increased, and *B. hypnorum* showed no clear response. Therefore, changes in the phenology of alpine plants due to global warming affect pollinators in highly species-specific ways.

**Supplementary Information:**

The online version contains supplementary material available at 10.1007/s00442-025-05775-4.

## Introduction

Global warming has influenced the phenological events of various organisms (Parmesan 2007; Ovaskainen et al. 2013). It often alters the synchrony (temporal overlap) between interacting species, such as prey and predators, plants and herbivores, flowers and pollinators, and fruits and frugivores (Renner and Zohner 2018). However, our knowledge of the mechanisms leading to phenological mismatch and its ecological significance is limited. Plant-pollinator interactions are one of the most fundamental mutualisms in terrestrial ecosystems (Ollerton et al. 2011). There are many reports of phenological shifts in both flowering plants and pollinator insects in response to ongoing global warming (Elzinga et al. 2007; Hegland et al. 2009; Inouye 2019; Stemkovski et al. 2020). There are also numerous studies analyzing phenological correlations between flowers and pollinators at the community scale, but many of these predict that the ecological significance of phenological mismatches in the pollination mutualism is limited due to similar phenological changes or generalized relationships between plants and pollinators (Hegland et al. 2009; Bartomeus et al. 2011; Rafferty and Ives 2011; Iler et al. 2013b; Renner and Zohner 2018; Gérard et al. 2020). Although phenological mismatch is expected to affect the fitness and population dynamics of both plants and pollinators (Memmott et al. 2007; Miller-Rushing et al. 2010; Iler et al. 2021), empirical studies demonstrating the ecological importance of phenological mismatch are limited (reviewed by Ogilvie and Forrest 2017; Gérard et al. 2020), and most of them have focused on the specialized interactions between specific species (e.g., Kudo and Ida 2013; Kehrberger and Holzschuh 2019; Weaver and Mallinger 2022). For example, a warm spring with early snowmelt causes a phenological mismatch between the spring ephemeral *Corydalis ambigua* and bumble bee queens, as flowering starts earlier than the emergence of bees from hibernation, resulting in low plant seed-set success due to pollen limitation (Kudo and Ida 2013; Kudo and Cooper 2019). However, even among studies of specialized interactions, the effects of phenological mismatch on pollinators have been poorly investigated (reviewed by Gérard et al. 2020).

In high-latitude and alpine ecosystems, warming temperature and decreasing snow cover period have affected the distributions and phenologies of plants and animals (Inouye 2019; Inouye and Wielgolaski 2013; Wielgolaski and Inouye 2013; Cooper 2014). Changes in the flowering phenology of arctic and alpine plants have been observed in long-term studies (e.g., Forrest et al. 2010; CaraDonna et al. 2014; Prevéy et al. 2019). As snow distribution in alpine ecosystems is extremely heterogeneous due to the complicated topography, flowering phenology of alpine plants is highly variable among communities and populations of individual species (Inouye 2019). The basic structure of alpine ecosystems is composed of windblown fellfield habitat with little snow cover and snowbed habitat with thick snow accumulation in winter, where flowering of alpine plants progresses from fellfield communities to snowbed communities in summer (Kudo 2016, 2019). Previous studies have shown that the flowering phenology of fellfield plants is influenced by the thermal conditions in early summer, but the extent of interannual variation in flowering is relatively small, whereas that of snowbed plants is strongly regulated by snowmelt timing, with large interannual variation (Hülber et al. 2010; Iler et al. 2013a; Petraglia et al. 2014; Kudo 2019). Warmer temperature and earlier snowmelt caused by global warming are expected to advance the flowering phenology of snowbed communities, and it may cause a shorter flowering season in alpine ecosystems, unless the warmer temperature extends flowering later into autumn (e.g., CaraDonna et al. 2014; Chen et al. 2023). Such a phenological shift should affect resources of flower visitors (Memmott et al. 2007; Rafferty and Ives 2011; Ogilvie et al. 2017; Inouye 2019).

Many alpine plants depend on insect pollinators for seed production (Kudo 2022), and bumble bees (*Bombus* spp.) are the most important pollinators in alpine ecosystems of the northern hemisphere (Pyke et al. 2011; Kudo 2019; Minachilis et al. 2020; Sposler et al. 2022). *Bombus* species that live in cold climates are generally sensitive to heat stress (Rasmont and Iserbyt 2012; Soroye et al. 2020). During recent decades, bumble bees have decreased in abundance worldwide and have shifted their distribution ranges in response to global warming (Ghisbain et al. 2024; Soroye et al. 2020) although responses are heterogeneous among species (Kerr et al. 2015). As climate change brings about various environmental changes in addition to warming temperature, such as drought stress, early snowmelt and spatiotemporal variation in floral resources for insects, its effects on bumble bee populations should be variable among species, habitat types, and regions.

Foraging activity of bumble bees shows a clear seasonality within a short summer in alpine regions, reflecting the annual life cycle; overwintered queens appear in early summer and start nesting, worker bees appear in mid-summer, and the active foraging period lasts until new queens and drones appear in late summer (Pyke et al. 2011; Kudo 2014). Reflecting the clear seasonality of worker bees, pollination success of bee-visited plants increases when flowering occurs in mid-summer (Kudo 2022). Early-flowering fellfield flowers are visited by overwintered queens, and late-flowering fellfield flowers are visited by worker bees, but the flowering period of fellfield sites often ends before the peak season of worker bees, and the main floral resources for worker bees are snowbed flowers. A preliminary report from our study area in Japan showed the occurrence of phenological mismatch in a summer with earlier snowmelt in which flowering progressed earlier than the peak abundance time of worker bees (Kudo 2014). However, little is known about the mechanism of phenological mismatch and its impact on population dynamics and species composition of bumble bees (but see Miller-Struttmann et al. 2022). The effects of phenological mismatch on bumble bee populations are expected to occur in the following year through reduced production of new queens when colony development is limited by available floral resources (Inari et al. 2012; Thomson 2016).

To evaluate the impact of climate change on bumble bees, therefore, it is important to analyze the effects of both increasing temperature and changes in the availability of floral resources caused by a shift in phenology. The impacts of phenological mismatch are expected to vary among bee species, depending on foraging behavior (breadth of flower choice), foraging distance from the nest, and life cycle (short or long colony lifespan). It is predicted that the negative effects of earlier and shorter flowering periods in alpine plant communities will be more serious for alpine-resident species with longer colony life-span than for species that visit alpine sites to forage or that have a short colony lifespan (Persson et al. 2015; Ogilvie and Forrest 2017; Miller-Struttmann et al. 2022). Furthermore, the effects of flowering shifts at the community level may vary among bee species, depending on the actual phenological shift of specific flowers.

This study aims to elucidate the following: (1) how alpine plant communities flower in response to variations in temperature and snowmelt, (2) the annual population dynamics of *Bombus* workers, (3) what causes the mismatch between the flowering of snowbed flowers and the emergence of worker bees, and (4) how this mismatch affects worker abundance. We expect the following patterns: (1) warmer temperatures and earlier snowmelt advance flowering time and shorten the flowering period; (2) interannual fluctuations in worker abundance vary between *Bombus* species, depending on their life cycles; (3) the phenological responses of alpine flowers to interannual climate variability are greater than those of bumblebees, leading to increased phenological mismatch between them; and (4) the effects of phenological mismatch on bumblebee population dynamics will be more severe for alpine-resident species than for species that visit alpine sites to forage.

## Materials and methods

### Study sites

Two observation sites of the long-term ecosystem monitoring project “Monitoring Sites 1000 (Biodiversity Center of Japan, URL: https://www.biodic.go.jp)” were established in the Taisetsu Mountains (Hokkaido, northern Japan) in 2010 on Mt. Kuro (Kuro site; 43.70°N, 142.92°E) and Mt. Aka (Aka site; 43.67°N, 142.93°E). These sites are 5.5 km apart. At each site, one fellfield plot and one snowbed plot (10 m × 20 m in size) were established for the monitoring of flowering phenology at community scale. The fellfield plots were located at 1960 and 1830 m a.s.l., and the snowbed plots located at 1890 and 1970 m a.s.l. at the Kuro site and the Aka site, respectively (Fig. S1). Air temperature at 1.5 m above the ground has been recorded at one-hour intervals at the Aka site at 1830 m elevation since 2010 using a thermistor thermometer (TR-52S) set in a solar-radiation shield (T and D Co. Ltd., Japan). The annual mean temperature during 2010–2023 was −2.7 °C, with monthly mean temperatures ranging from −17.6 °C (January) to 12.6 °C (July). The fellfield plots are exposed during the winter with little snow cover and soil is frozen from October to May. The snowbed plots are covered with thick snow during the winter and snow remains until mid-summer, but actual snowmelt time varies highly among years. Snowmelt in the snowbed plots usually occurs one-week earlier at the Kuro site (29 June in average, ranging from 13 June to 10 July) than at the Aka site (5 July, from 21 June to 25 July).

### Data sets of flowering phenology

During the growing season from June to September, flowering stages of entomophilous plant species were recorded at 2–3 days intervals on average by citizen volunteers. As in a previous report on this study system (Kudo 2019), plant species in the fellfield plots were classified into two groups, early bloomer and late bloomer, in which flowering of the former group occurred from early June to early July and that of the latter group occurred after mid-July. Plant species growing in the snowbed plots were classified as snowbed plants, with flowering typically occurring from mid-July to late August, but the actual flowering season varied greatly from year to year. Thus, the flowering patterns of alpine plants are composed of flowering sequences of fellfield early-bloomer (FE), late-bloomer (FL) and snowbed plants (S). There was some overlap of species between the fellfield plots and the snowbed plots (see Table S1). As bumble bee workers usually appear after mid-July, the main floral resources for workers are FL and S plants.

The description of flowering phenology and the analyses are conducted following the procedure of Kudo (2019). Flowering stages of individual species were qualitatively categorized at four classes (A, B, C, D) as follows: A is the beginning of flowering, B is the peak flowering, C is the middle to late flowering period, and D is the end of flowering season. The main flowering period of each species is defined from the first record of stage A to the last record of stage C in each plot. Flowering progress of each group (FE, FL, S) was expressed as the number of flowering species (stages A to C) in each plot. The seasonal progress of the number of flowering species within a plot in each year was fitted by a generalized linear model (GLM) with log-link Poisson error distribution of unimodal structure (i.e., inclusion of a quadratic term of day) as follows: y = exp (*a* + *b* x + *c* x^2^), where *a*, *b*, c are coefficients and x is observation day. All analyses were performed on R ver. 4.4.2 (R Core Team 2024). Fitting of the GLMs was checked by Nagelkerke’s R^2^ using the ‘performance’ package. We confirmed that the flowering progress of each group fitted the unimodal model in each year at both sites (*P* < 0.0001, R^2^ = 0.892–0.999).

**Fig. 1:**
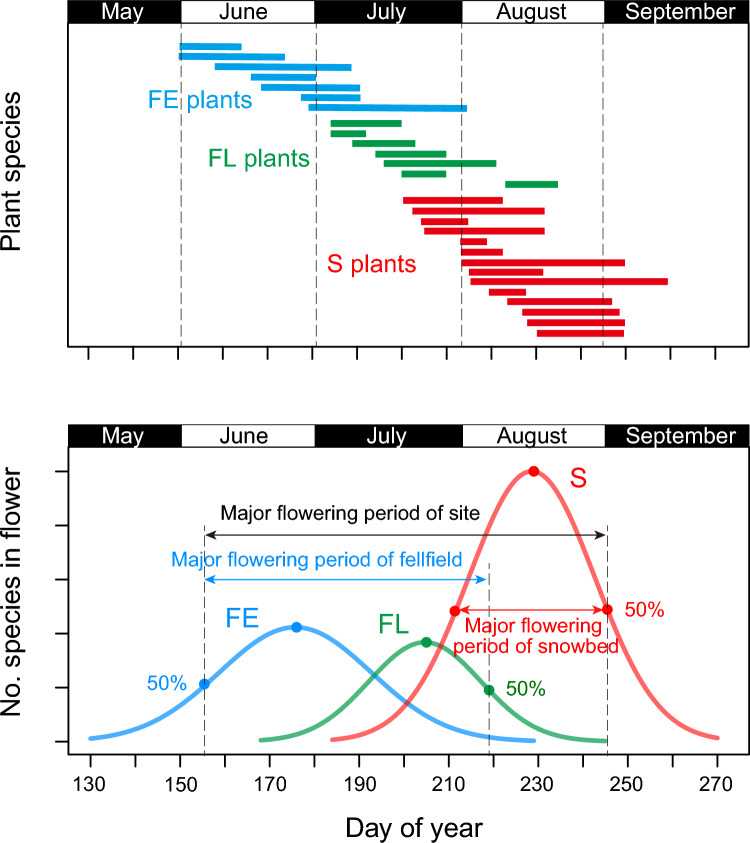
**a** An example of flowering sequence of early (FE) and late flowering species (FL) in the fellfield community and species in the snowbed community (S) at the Aka site. **b** Calculation of flowering periods of plant communities from the qualitative phenological record

Based on the fitted function in each of FE, FL, and S group, we estimated the peak flowering day and the major flowering period of each group in each year, in which peak flowering day means the day when the number of flowering species reached a peak, and major flowering period means the duration between the days when flowering of 50% species was observed in early and late flowering season, respectively (Fig. 1). From the estimated values, the major flowering period of a whole site is expressed as the duration from the early 50% flowering day of EF to the late 50% flowering day of S at each site.

The limitation of our flowering phenology data is that floral resources are recorded qualitatively, i.e., the number of flowering species, rather than numbers of flowers. This is because our baseline flowering phenology data were collected by citizen volunteers, for whom a simple, straightforward protocol was recommended for field surveys. As a complement to these data, we have included quantitative data on seasonal changes in the floral abundance of bee-visited plants (conducted in 2020) as a point of reference (see Fig. S2).

### Factors affecting flowering phenology

The responses of flowering phenology (peak flowering day and major flowering period) of each group to temperature (for all groups) and snowmelt time (only for S) were analyzed using linear models. For FE plants, the explanatory variables were mean May and June temperatures (measured at the Aka site) of each year (deviations from the mean temperature during observed period, 2010–2023) and site (Kuro, Aka), with interaction terms between temperature and site. The same models were performed for FL plants, but mean May, June, and July temperatures are included in the models. For S plants, mean June, July, and August temperatures, snowmelt time (deviation from the mean value), and site were included as the explanatory variables with interaction terms between site and other factors. Furthermore, the response of major flowering period within sites to temperature and snowmelt time was tested by a linear model, where the explanatory variables were mean May, June, July and August temperatures, and snowmelt time. For each model, best-fit selection of explanatory variables was performed based on the AICc using the ‘MuMIn’ package on R ver. 4.4.2.

### Data set of bumble bee observation

We observed bumble bees along the fixed trails at the Kuro (2012–2023) and Aka (2011–2023) sites. The census trail at the Kuro site was 2.0 km long, ranging in elevation from 1520 to 1984 m, and the census trail at the Aka site was 3.4 km long, ranging in elevation from 1490 to 1845 m (see Fig. S1). These trails were selected to connect the flowering phenology monitoring plots at each site. Observers walked slowly along the trail, recording bumble bees foraging on flowers within a distance of about 2.5 m on both sides of the trail (i.e., a width of 5 m). Each trail was divided into two or three intervals (Fig. S1), but pooled data from each census within a site were used for analysis in this study. Observed bees were identified to species and caste (workers, queens, drones) if possible. In these sites, common bumble bee species (*Bombus* spp.) are *B. hypocrita sapporoensis*, *B. beaticola moshkarareppus*, and *B. yezoensis*, while *B. hypnorum koropokkrus* is sometimes observed but at lower frequency. In contrast, *B. diversus tersatus* and the exotic *B. terrestris* are very rare and excluded from the analyses (see Table 1).

**Table 1:** Number of individual bumble bee workers of each species (*Bombus* spp.) recorded during the study period (2012–2023) in the Kuro site and the Aka site

*Bombus* species	Kuro site	Aka site
*B. hypocrita sapporoensis*	2126 (13.6%)	2915 (40.2%)
*B. beaticola moshkarareppu*	5347 (34.1%)	2140 (29.5%)
*B. yezoensis*	7746 (49.5%)	1,440 (19.8%)
*B. hypnorum koropokkrus*	330 (2.1%)	760 (10.5%)
*B. diversus tersatus*	102 (< 0.01%)	4 (< 0.001%)
*B. terrestris*	10 (< 0.001%)	1 (< 0.001%)
Total	15,661	7260

*Bombus hypocrita* is the most common bumble bee species in the alpine ecosystems of Hokkaido. Although this species has a wide distribution range, from coastal to alpine regions, alpine populations reside in the alpine area throughout their life cycle, i.e., their overwintering, nesting and foraging sites are all in the alpine zone. *Bombus beaticola* is also common in alpine environments, with all castes (queens, workers and males) observed, but the overwintering sites of queens may be in the forest zone, as queens are rare in alpine areas early in the season. In contrast, *B. yezoensis* and *B. hypnorum* are visitors in the alpine zone; mainly worker bees come to the alpine zone for foraging, although queens and drones are sometimes observed at the study sites (Kudo 2014). In the interior part of the Taisetsu Mountains (more than 2 km from the forest zone), only *B. hypocrita* and *B. beaticola* are common, while other *Bombus* species are rare (Mizunaga and Kudo 2017). This suggests that the frequency with which alpine-visiting bumble bees appear depends on how accessible the alpine habitat is from low-elevation habitats.

Throughout the observation periods, more than 95% of the bumble bees observed were identified at species level. Therefore, unidentified bees were excluded from the analyses. The observation period was most intensive from early July to early September (about once a week), in order to cover the entire active season of worker bees, but observations were also conducted irregularly in June. Due to unstable weather conditions in the alpine area, data collected on foggy, windy or rainy days were excluded from the analyses. The mean number of observation days per year was 11.5 (ranging from 8 to 14 days) at the Aka site, and 9.7 (ranging from 8 to 12 days) at the Kuro site.

As reported in a previous study of montane bumble bees (Pyke et al. 2011), the frequencies of worker bees of individual *Bombus* species exhibited a unimodal pattern with seasonal progress from mid-July to early September. Thus, the seasonal dynamics of the worker bees were modelled using a GLM with a log-link Poisson error distribution, which had a unimodal structure at each site and in each year, as mentioned before. The day on which the maximum value was detected by the GLM corresponded to the peak day of worker activity, and the predicted value represented the peak abundance of worker bees of each species at each site and in each year.

### Phenological mismatch between flowering time and worker bees

As the active period of worker bees often overlaps with the flowering period of snowbed communities (S plants), we focused on the difference between the peak flowering time of S plants and the peak appearance time of worker bees of each *Bombus* species. In the present study, phenological mismatch is defined as the time difference (in days) between the peak flowering of S plants and the peak abundance of worker bees at each site, i.e., the time lag between worker activity and the peak of flowering of S plants in the snowbed habitat. A positive value indicates a delay in the appearance of worker bees, while a negative value indicates a delay in flowering. When worker bees appear before the peak flowering of snowbed communities (i.e., when there is a negative mismatch value), they can forage in fellfield communities. However, when they appear after the peak flowering of snowbed communities (i.e., a positive mismatch value), the available floral resources may be limited, which can have negative impacts on colony development and subsequent population dynamics. The environmental factors affecting phenological mismatch were analyzed using linear models for individual *Bombus* species, where the predicted variable was phenological mismatch (the difference between the peak day of the worker bees and the peak flowering day of the S plants) and the explanatory variables were the deviations in snowmelt time and mean temperatures during early and mid-summer, with interaction terms for the sites. The best-fit model was selected based on the AICc values, as mentioned before.

### Factors affecting bumble bee activity

The influence of environmental factors on the peak appearance day of worker bees was analyzed for each *Bombus* species using a linear model, where the response variable was the peak appearance day of worker bees, and the explanatory variables were snowmelt day and mean air temperatures during early summer (June) and mid-summer (July and August) of the current year, including interaction terms with site (Kuro and Aka sites). Early summer corresponds to the nesting period of overwintered queen bees, while mid-summer corresponds to the active period of worker bees. Deviations from the mean values during the years of observation were used for the snowmelt day and temperatures in early and mid-summer.

In the analysis of the peak abundance of worker bees in each *Bombus* species, a GLM with a negative binomial error distribution was used to reduce overdispersion (using the *glm.nb* function in the ‘MASS’ package on R ver. 4.4.2) in which the response variable was peak abundance of worker bees and the explanatory variables were snowmelt day, temperatures in the current season (early and mid-summer) and phenological mismatch in the previous year, including interaction terms with site. Explanatory variables were selected for each model based on AICc values for the linear model or AIC values for the GLM. All drawing figures of the statistical results were generated using the ‘ggplot2’ package on R ver. 4.2.2.

## Results

### Flowering phenology

Throughout the observation period (2010–2023), the flowering of 7, 10 and 14 species of FE, FL and S plants was detected at the Kuro site, and the flowering of 8, 8 and 17 species was detected at the Aka site (see Table S1).

**Fig. 2:**
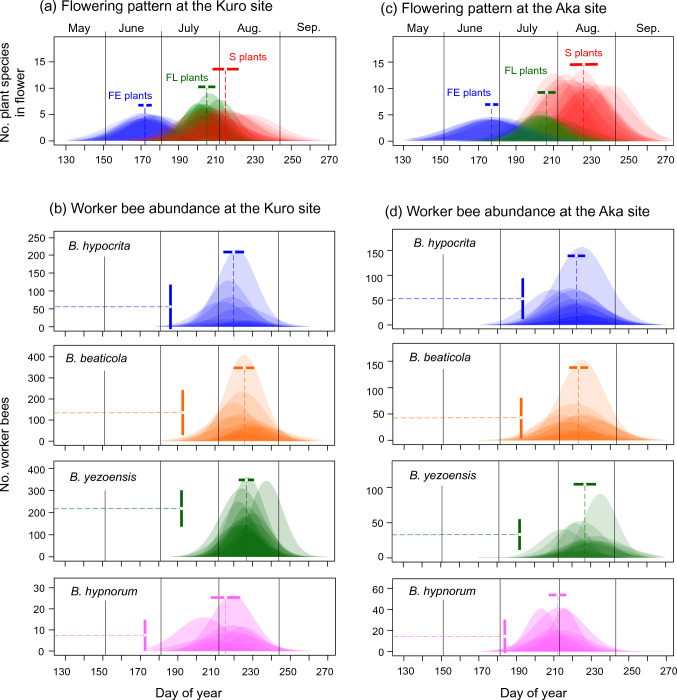
Upper panels indicate the yearly variations (2010–2023) in the flowering sequences of EL plants, FL plants, and S plants at the Kuro (**a**) and Aka (**c**) sites. Mean flowering peak day and standard deviation in each group are shown. Lower panels indicate the yearly variations (2012–2023) in the seasonal dynamics of worker bees of major *Bombus* species at the Kuro (**b**) and Aka (**d**) sites. Peak abundance day (horizontal bar) and the maximum abundance of each species (vertical bar) are shown with standard deviation

The flowering sequences of the FE, FL and S plants remained consistent throughout the monitoring period (2010–2023) at each site (Fig. 2a, c). Yearly variations in flowering phenology were smallest in the FE plants and largest in the S plants. Flowering of the FE plants began in late May (148.4 ± 3.7 SD at the Kuro site and 146.9 ± 4.7 SD at the Aka site, on the day of the year) and peaked in late June (172.6 ± 3.7 and 177.1 ± 3.5, respectively). Flowering of the FL plants began at the beginning of July (187.1 ± 6.0 at the Kuro site and 183.1 ± 5.1 at the Aka site) and peaked in late July (204.6 ± 4.9 and 206.4 ± 4.9, respectively). The major flowering period of the fellfield community was 60.3 ± 4.1 days at the Kuro site and 66.8 ± 5.1 days at the Aka site. The flowering onset of the S plants occurred in mid-July (187.3 ± 8.3 at the Kuro site and 197.1 ± 8.8 at the Aka site) and peaked in early August (214.6 ± 7.3 and 226.4 ± 7.6, respectively). The major flowering period of the snowbed community was 36.1 ± 2.4 days at the Kuro site and 32.7 ± 3.2 days at the Aka site. The major flowering period for each site (combining the fellfield and snowbed communities) was 77.1 ± 7.0 days at the Kuro site and 87.4 ± 7.2 days at the Aka site.

The best-fit model predicts that the peak flowering time of FE plants occurs earlier when May and June are warm. With 1 ºC of warming, the peak flowering time occurs 3.2 days earlier (1.7 days and 1.5 days earlier with warming in May and June, respectively). The major flowering period is also shorter when June is warm (1.6 days shorter with 1 °C of warming; Table 2a). Similarly, the peak flowering time of FL plants occurs earlier when May and June are warm, with 1 °C of warming causing a 4.6-day earlier peak flowering time (1.6 days and 3.0 days earlier with warming in May and June, respectively). However, only May temperature moderately affects the major flowering period (1.4 days shorter with 1 °C of warming; Table 2b). The major flowering period of the fellfield community (FE + FL) is predicted to be 3.6 days shorter with 1 °C of warming in June and July (1.9 days and 1.7 days shorter, respectively; however, the effect of July temperature was not significant; Table 2d). The peak flowering time of the snowbed community (S plants) is influenced by June and August temperatures and snowmelt time (Table 2c). With 1 °C of warming, flowering occurs 1.9 days earlier (with 0.9 and 1.1 days shorter flowering periods in June and August, respectively), while 10 days earlier snowmelt causes flowering to occur 7.6 days earlier. Therefore, the peak flowering of the snowbed community will occur 9.5 days earlier with 1 °C of warming and 10 days earlier snowmelt. In contrast, neither temperature nor snowmelt time affects the flowering period of the snowbed community.

**Table 2:** The results of linear models fitted to the flowering phenologies (peak flowering day and major flowering period) of FE plants (a), FL plants (b), S plants (c), and the major flowering periods of the fellfield community (d) and the whole site from fellfield to snowbed communities (e)

	Coeff	SE	*t* value	*P* value
(a) Phenology of FE plants				
Peak flowering time AICc = 140.0 (155.0)				
Intercept (Kuro site)	172.8	0.68	254.4	< 0.0001***
ΔMay temp	−1.73	0.48	−3.60	0.0014**
ΔJune temp	−1.51	0.43	−3.55	0.0016**
Aka site	4.36	0.96	4.54	0.0001***
Flowering period AICc = 153.5 (170.0)				
Intercept (Kuro site)	32.93	0.89	36.9	< 0.0001***
ΔJune temp	−1.61	0.47	−3.38	0.0024**
Aka site	9.29	1.26	7.34	< 0.0001***
(b) Phenology of FL plants				
Peak flowering time AICc = 142.5 (155.0)				
Intercept	204.8	0.52	394.2	< 0.0001***
ΔMay temp	−1.63	0.52	−3.15	0.0042**
ΔJune temp	−2.96	0.46	−6.43	< 0.0001***
Flowering period AICc = 161.9 (174.8)				
Intercept (Kuro site)	21.67	1.04	20.9	< 0.0001***
ΔMay temp	−1.37	0.62	−2.21	0.036*
Aka site	9.00	1.46	6.15	< 0.0001***
(c) Phenology of S plants				
Peak flowering time AICc = 144.5 (162.5)				
Intercept (Kuro site)	212.9	0.71	300.9	< 0.0001***
ΔJune temp	−0.85	0.39	−2.20	0.038*
ΔAugust temp	−1.09	0.35	−3.11	0.0049**
ΔSnowmelt time	0.76	0.06	13.4	< 0.0001***
Aka site	10.77	1.00	10.8	< 0.0001***
Flowering period AICc = 163.5 (182.9)				
Intercept (Aka site)	35.14	1.10	32.0	< 0.0001***
Aka site	−2.71	1.55	−1.75	0.092^+^
(d) Flowering period of fellfield plot AICc = 173.9 (184.5)				
Intercept (Kuro site)	58.39	1.24	47.0	< 0.0001***
ΔJune temp	−1.91	0.66	−2.89	0.00080**
ΔJuly temp	−1.67	0.99	−1.68	0.104
Aka site	6.29	1.76	3.58	0.0015**
(e) Whole flowering period in site AICc = 183.2 (210.9)				
Intercept (Kuro site)	74.07	1.36	54.6	< 0.0001***
ΔMay temp	1.68	0.87	1.94	0.065^+^
ΔJuly temp	−2.24	1.19	−2.20	0.073^+^
ΔAugust temp	−2.21	0.71	−3.10	0.0053**
ΔSnowmelt time	0.64	0.11	5.84	< 0.0001***
Aka site	9.92	1.91	5.18	< 0.0001***

The best-fit model of the major flowering period for the entire site, from fellfield to snowbed communities, predicts that the warming effect varies by month (Table 2e). A warm May, for example, will lengthen the flowering period by 1.7 days with 1 °C of warming, whereas warm July and August will shorten the flowering period by 2.2 days. Combining 1 °C of warming with 10 days of earlier snowmelt is predicted to shorten the overall flowering period by 9.2 days.

### Peak abundance time of bumble bee workers

A total of 15,661 and 7260 worker bees were recorded at the Kuro site and the Aka site, respectively, throughout the 12-year monitoring period (2012–2023) during which bee observations were conducted at both sites (Table 1). The dominant species at the Kuro site were *B. yezoensis* (49.5%) and *B. beaticola* (34.1%), while at the Aka site they were *B. hypocrita* (40.2%) and *B. beaticol*a (29.5%). *Bombus diversus* and the non-native *B. terrestris* were very rare (< 0.01%). As previously mentioned, these two species were excluded from the analyses in this study.

The annual variation in the peak time of worker bees was smaller for each *Bombus* species than the variation in the peak flowering time of the snowbed communities (Fig. 2b, d). GLM results showed no significant differences in peak times between sites, and snowmelt time and early and mid-summer temperatures were excluded by AIC model selection or had little effect on the peak abundance times in each species (*P* > 0.05; Table S2). These results suggest that the phenologies of bumble bees are relatively invariant.

### Environmental factors causing phenological mismatch

**Fig. 3:**
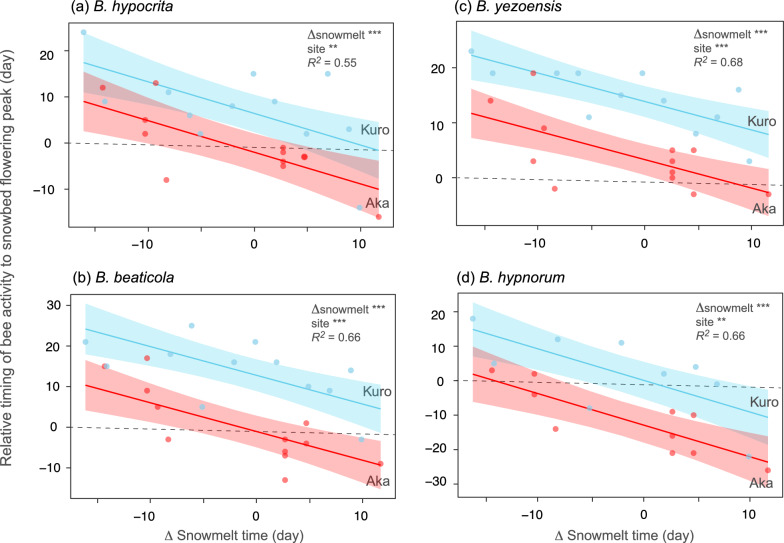
Relationships between snowmelt time and the phenological mismatch between worker bees and snowbed flowers, i.e., the time lag of worker activity relative to the peak of flowering in snowbed community for major *Bombus* species at the Kuro and Aka sites. A positive value means a delay in the appearance of worker bees. Refer to Table S3 for statistical details

Analyses of the phenological mismatch between the peak flowering of snowbed plants and the peak abundance time of worker bees showed that early snowmelt was associated with an increasing delay in bee activity peaking relative to snowbed flowering (Table S3; Fig. 3). The mismatch period was longer at the Kuro site, where snowmelt occurred approximately one week earlier than at the Aka site. Summer temperatures (both early and mid-summer) were not related to phenological mismatch in any *Bombus* species. Therefore, the phenological mismatch between snowbed flowers and worker bees was primarily influenced by the timing of snowmelt in the current season.

### Population dynamics of bumble bee workers

**Fig. 4:**
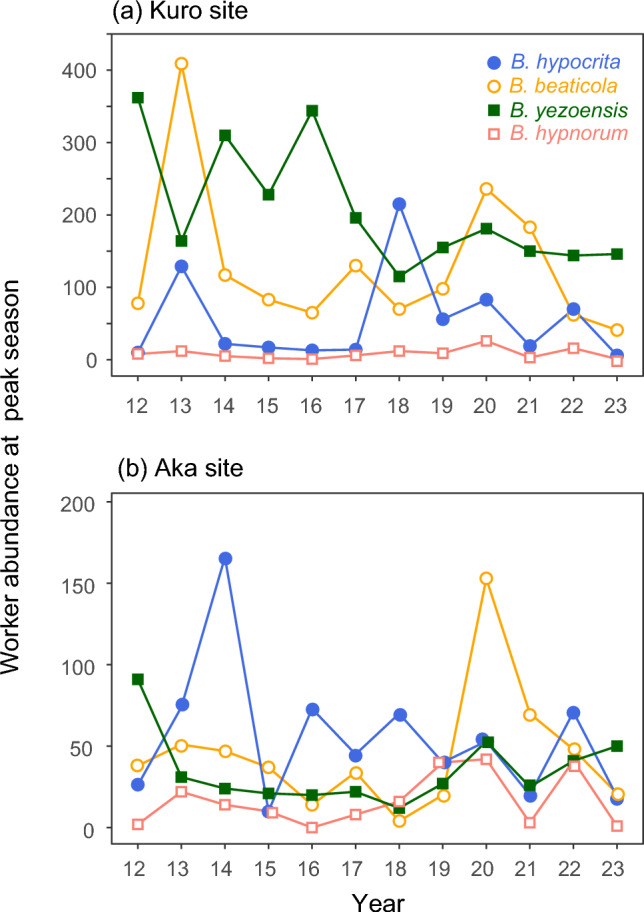
Annual variation in peak abundance of worker bees of four *Bombus* species from 2012 to 2023 at the Kuro (**a**) and Aka (**b**) sites

In contrast to the small yearly variations in the peak abundance time of worker bees, the number of worker bees during the peak season fluctuated greatly from year to year in every *Bombus* species (Fig. 2b, d). Population dynamics patterns varied distinctly among species and between sites even in the same species (Fig. 4). For instance, *B. hypocrita* increased in 2013 and 2018 at the Kuro site but only in 2014 at the Aka site; *B. beaticola* increased in 2013 and 2020 at the Kuro site but only in 2020 at the Aka site; *B. yezoensis* increased in 2012, 2014 and 2016 at the Kuro site but only in 2012 at the Aka site.

**Fig. 5:**
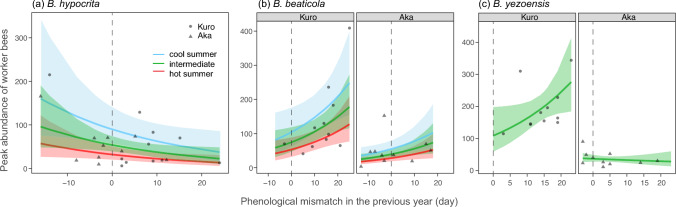
Relationships between phenological mismatch in the previous year and the peak abundance of key *Bombus* workers at each site. Data points show the model-predicted values for each years. Blue, green and red curves indicate the patterns in cool, medium and warm summers in the current year, as modelled by the GLMs (see Table 3 for statistical details). Of four major *Bombus* species, data of *B. hypnorum* is not shown, as the worker abundance of this species was independent of previous phenological mismatch. There was no significant difference in worker abundance between sites for *B. hypocrita*. For *B. yezoensis*, worker abundance was independent of summer temperatures

The GLM results suggest that the factors influencing worker population dynamics are strongly species-specific (Table 3). For *B. hypocrita*, mid-summer temperature (July and August) and phenological mismatch in the previous year were negatively correlated with worker abundance. Meanwhile, snowmelt time, early summer temperature (June) and site were excluded as explanatory variables by AIC model selection (Fig. 5a, Table 3a).

**Table 3:** The results of negative binomial GLMs fitted to the peak abundance of worker bees of the major *Bombus* species

	Coeff	SE	*z* value	*P* value
(a) *B. hypocrita* AIC = 206.4 (217.9)				
Intercept	3.940	0.162	24.38	< 0.0001***
ΔMid-summer temp	−0.617	0.196	−3.15	< 0.0017**
Previous mismatch	−0.036	0.017	−2.16	0.031*
(b) *B. beaticola* AIC = 221.8 (227.5)				
Intercept (Kuro site)	4.337	0.245	17.70	< 0.0001***
ΔSnowmelt time	0.016	0.018	0.88	0.38
ΔEarly summer temp	0.296	0.098	3.02	0.0026**
ΔMid-summer temp	−0.401	0.142	−2.83	0.0047**
Previous mismatch	0.035	0.014	2.44	0.015*
Aka site	−0.789	0.278	−2.84	0.0045**
ΔSnowmelt × Aka	−0.077	0.028	−2.75	0.0060**
(c) *B. yezoensis* AIC = 214.9 (221.8)				
Intercept (Kuro site)	4.639	0.313	14.83	< 0.000 ***
ΔSnowmelt time	−0.022	0.011	−2.02	0.043*
Previous mismatch	0.041	0.020	2.03	0.043*
Aka site	−1.061	0.327	−3.24	0.0012**
Previous mismatch × Aka	−0.054	0.026	−2.08	0.038*
(d) *B. hypnorum* AIC = 146.5 (155.7)				
Intercept (Kuro site)	2.162	0.123	17.54	< 0.0001***
ΔMid-summer temp	−0.398	0.181	−2.20	0.028*
Aka site	0.739	0.184	4.01	< 0.0001***

For *B. beaticola*, mid-summer temperature was negatively correlated with worker abundance, whereas early-summer temperature and previous phenological mismatch were positively correlated with worker abundance. Worker abundance was higher at the Kuro site than at the Aka site. A significant negative interaction was observed between snowmelt time and site, suggesting that the rapid progress of flowering caused by earlier snowmelt would benefit *B. beaticola* at the Aka site, where snowmelt progresses slowly (Fig. 5b, Table 3b).

The abundance of *B. yezoensis* workers was much higher at the Kuro site than at the Aka site. Both early and mid-summer temperatures were excluded by AIC model selection. Worker abundance was negatively influenced by snowmelt time at both site, suggesting that *B. yezoensis* would also benefit from the rapid progress of flowering caused by earlier snowmelt. There was a significant negative interaction between previous phenological mismatch and site, meaning that previous phenological mismatch was only positively correlated with worker abundance at the Kuro site (Fig. 5c, Table 3c).

The abundance of *B. hypnorum* workers was much higher at the Aka site than at the Kuro site. The peak abundance of worker bees was negatively related to mid-summer temperature, while AIC model selection excluded other environmental factors and previous phenological mismatch (Table 3d).

## Discussion

Our study showed that warmer temperature and earlier snowmelt significantly affected the flowering phenology of alpine plant communities, with an advancing phenology of snowbed plants shortening the overall flowering season in the alpine region. In contrast, the phenology of bumble bees was less sensitive to weather and snowmelt conditions across years, suggesting that their life cycle is more conservative. Consequently, the phenological mismatch is expected to accelerate with global warming, as bumble bees will be unable to keep up with the advancing flowering phenology. The ecological consequences of phenological mismatch are predicted to vary between *Bombus* species.

### Phenological responses of alpine plants and bumble bees

The trends of phenological dynamics of alpine plant communities obtained in this study (2010–2023) were broadly consistent with those obtained in a previous analysis of nine years of phenological records (2010–2018) at the same sites (Kudo 2019). The flowering phenology of fellfield communities advanced in response to warmer temperature early in the season, whereas the flowering phenology of snowbed communities was strongly regulated by snowmelt time. In the fellfield communities, the rate of flowering advance was lower for early-flowering plants than for late-flowering plants. Consequently, the overall flowering season in alpine plant communities is expected to shorten if climate change brings both warmer temperature and earlier snowmelt. A similar trend in community-scale flowering patterns has also been reported in tundra ecosystems (Prevéy et al. 2019). Our model predicts that the overall flowering period of alpine plant communities will shorten by 9.2 days with 1 °C of warming and 10 days earlier snowmelt, corresponding to a 11–12% decrease in the total flowering period. This is twice the size of the previous prediction (4.8 days shorter, Kudo 2019). This difference may be due to the inclusion of more recent data (2019–2023 records), during which the summer temperature was higher and the snowmelt occurred earlier than in previous years.

The habitat-specific responses of flowering phenology to environmental factors may reflect the selective forces operating within each habitat. As the risk of frost damage is higher during the early growing season in the fellfiled habitat (Inouye 2008; Rixen et al. 2012), a conservative flowering habit that reduces the risk may be beneficial, particularly for early bloomers. In the snowbed habitat, plants are exposed from snow cover in midsummer, where initiating growth and flowering soon after snowmelt is beneficial in order to complete the life cycle during the short summer (Cooper et al. 2011; Petraglia et al. 2014; Kudo 2016). Therefore, consideration of heterogeneous snow distribution is crucial for predicting the phenological responses of alpine plant communities at a landscape level (Forrest et al. 2010; Hülber et al. 2010; Iler et al. 2013a; Kudo 2016).

Unlike flowering phenology, the phenology of worker bees was independent of temperature and snowmelt time, suggesting a consistent life cycle for *Bombus* species. A similar trend was observed in the Rocky Mountains, where the phenology of worker bees remained unchanged between 1974 and 2007 (Pyke et al. 2016). Specifically, the phenology of worker bees of alpine resident species showed less variance than that of colonizing species from lower elevations (Miller-Struttmann et al. 2022). Conversely, queen emergence tends to occur earlier in warmer springs with early snowmelt (Kudo and Ida 2013; Koppel and Kerr 2022; Blasi et al. 2023). Several studies have reported an earlier emergence time for solitary bees in warmer springs (Bartomeus et al. 2011; Forrest and Thomson 2011; Stemkovski et al. 2020; Weaver and Mallinger 2022). These differences in phenological responses among bees reflect life-history-specific sensitivities: spring phenology is more sensitive to warming effects than summer phenology; insects with different overwintering sites (above- vs. belowground) experience different conditions and rely on different environmental cues for diapause termination; and social bees tend to exhibit more conservative phenology than solitary bees (Ogilvie and Forrest 2017), probably because colonies require time to develop.

Even when flowering and bee phenologies are both determined by the same environmental factors such as temperature, flowering phenology is generally more sensitive to climate variation than bee phenology. This results in a phenological mismatch, with flowering occurring earlier than the peak abundance of bees (Forrest and Thomson 2011; Kudo and Ida 2013; Kehrberger and Holzschuh 2019; Stemkovski et al. 2020; Miller-Struttmann et al. 2022). Snowbed communities provide important floral resources for worker bees, but the flowering periods of snowbed communities are highly variable from year to year due to large variations in snowmelt timing (Kudo 2014). Therefore, the phenological mismatch between the flowering of alpine plants and the activity of worker bees is regulated by the dynamics of the snowmelt regime rather than simply by warmer temperatures.

### Ecological significance of phenological mismatch for bumble bees

Unlike the stable phenology of worker bees across years, their abundance at peak season varied greatly from year to year, and patterns of population dynamics were also inconsistent between *Bombus* species at the same sites and between sites (Fig. 4). These species-specific dynamics suggest that the factors influencing worker abundance also vary between species. Several studies have demonstrated a strong relationship between floral resource levels and the abundance of bumble bees (Inari et al. 2012; Crone and Williams 2016; Thomson 2016; Ogilvie et al. 2017). Climate-driven changes in flowering phenology in alpine communities are also predicted to affect bee abundance (Aldridge et al. 2011; CaraDonna et al. 2014). While phenological mismatch limits the availability of accessible resources for bumble bees, no study has examined the impact of phenological mismatch between alpine plants and bumble bees on bee abundance in natural environments. Limited floral resources during colony development should reduce the production of new queens, which may impact population dynamics in the following season (Inari et al. 2012). Our analyses revealed significant variation in the responses of worker abundance to phenological mismatch in the previous year among *Bombus* species (Fig. 5). This variation may reflect the life cycles and resource use patterns of the different species. Ogilvie et al. (2017) also found differences in the responses of montane bumble bee species to variation in floral resources. Therefore, it may not be possible to generalize the effects of variation in floral resources across all *Bombus* species.

Among the four *Bombus* species, a negative effect of phenological mismatch was only observed in *B. hypocrita*, a typical alpine resident, where the abundance of workers consistently decreased with phenological mismatch in the previous year. This pattern supports the prediction that mismatch between the flowering of snowbed plants and the emergence of worker bees reduces bee abundance by limiting floral resources. The foraging range of high-elevation nesting bumble bees has been reported to be smaller (mostly 25–100 m) than that of lowland bumble bees (Elliott 2009; Geib et al. 2015). Consequently, the negative effects of a shorter flowering period caused by phenological mismatch could be more severe for alpine-resident bumble bees.

In contrast, the positive effects of phenological mismatch were observed in *B. beaticola* and *B. yezoensis*, with this trend being more pronounced at the Kuro site. These bumble bees are long-tongued species that prefer to visit thistle flowers (Asteraceae) with long floral tubes, such as *Cirsium kamtschaticum*, *Cirsium yezoalpinum*, and *Saussurea riederi* subsp. *yezoensis*. As these species tend to flower late in the season, usually after the peak flowering of the snowbed community (see Fig. S2), their flowering periods overlap with the peak abundance of worker bees, which occurs when flowering progresses rapidly due to earlier snowmelt. Of these plants, *C. kamtschaticum* was particularly abundant in the lower alpine grassland at the Kuro site. Thus, the positive correlation between advancing flowering phenology at the community level and the abundance of *B. beaticola* and *B. yezoensis* workers reflects the phenological matching between the bees and their preferred flowers. As *B. hypocrita* is a short-tongued species, visits to long-tube flowers are less frequent.

Finally, *B. hypnorum* has the shortest colony life-span among the four *Bombus* species, with the abundance of worker bees reaching its maximum in early August. As *B. hypnorum* workers can visit both fellfield and snowbed flowers, the interannual variation in the flowering phenology of snowbed plants may not significantly affect the population dynamics of this species. Therefore, the impact of phenological shifts in alpine plant communities varies among *Bombus* species, depending on their life cycle and foraging behavior in relation to morphological traits. Our results highlight the importance of considering species-specific variations in life cycles and foraging behaviors when assessing the ecological significance of phenological mismatch (Renner and Zohner 2018; Gérad et al. 2020).

### Abiotic factors affecting bumble bee abundance

Warmer mid-summer temperatures were associated with lower number of workers for three *Bombus* species, with the effect being more pronounced for the alpine-resident species, *B. hypocrita* and *B. beaticola* (Table 3). A similar trend was reported in the central Rocky Mountains, where the abundance of resident *Bombus* workers decreased with increasing summer temperature, though this was not observed for colonizing *Bombus* species in alpine sites (Miller-Struttmann et al. 2022). This suggests that hot summers may reduce worker foraging activity, particularly for cold-adapted species, due to physiological heat stress (Oyen et al. 2016; Soroyo et al. 2020). In contrast, early summer temperature had no effect on worker abundance for three *Bombus* species, and had a positive effect on *B. beaticola*. June is the nesting season for alpine species, when the mean temperature is much lower (7.9 °C in June) than in midsummer (12.3–12.6 °C in July–August) at the study site (Aka site, 1840 m a.s.l.; monthly mean during 2010–2023). Warm and stable nest temperatures are essential for successful colony development in cool environments (Goulson 2009; Gradisek et al. 2023). Therefore, warm temperatures during the nesting season may not be harmful, and may even be beneficial, for bumble bees nesting in alpine habitats.

The snowmelt time of the current year significantly affected the worker abundance of. *B. beaticola* and *B. yezoensis*. Earlier snowmelt could advance flowering phenology, resulting in a higher overlap period between worker bees of these species and late-blooming thistle flowers (see Fig. S2). This could lead to higher worker abundance. Therefore, the current floral resources, as well as those from the previous year, may be an important driver of bee abundance (Oglivie et al. 2017). However, as our analyses are based on the flowering phenology of typical plant communities (fellfield and snowbed communities), it is difficult to quantify the effects of a phenological shift on resource availability for each *Bombus* species. To further elucidate the species-specific response of bee abundance to a phenological shift, the relationships between the floral resources of key plant species and the foraging patterns of each *Bombus* species must be clarified.

## Concluding remarks

Bumble bees, which have an annual colony development cycle, are expected to be vulnerable to changes in flowering phenology in alpine ecosystems. However, response patterns varied widely between *Bombus* species and sites. This suggests that the effects of global warming on plant-pollinator interactions through phenological changes will vary regionally in alpine ecosystems. These variations reflect the morphological fit between pollinators and flowers (e.g., tongue and flower tube lengths), the species composition of local plant communities (i.e., floral diversity) and the accessibility of alpine habitats to migrant pollinators (i.e., pollinator diversity). Further studies on the predicted impacts of climate change and the conservation of high mountain ecosystems are crucial to further clarify the links between phenological shifts and population dynamics of interacting species.

## Supplementary Information

Below is the link to the electronic supplementary material.

Supplementary file1 (PDF 794 KB)

## Data Availability

The datasets used and/or analyzed during the current study are available from the corresponding author on reasonable request.
