# Supplementary material for: Phenological mismatch between alpine flowers and bumble bees: its mechanism and impacts on the population dynamics of bumble bees
Source: Oecologia. 2025 Aug 23;207(9):150. doi: 10.1007/s00442-025-05775-4 (PMC12374912; doi:10.1007/s00442-025-05775-4)
Supplement: Supplementary file 1 — Supplementary file1 (PDF 794 KB) [file 442_2025_5775_MOESM1_ESM.pdf]

**Electronic Supplemental Material (ESM)**

Phenological mismatch between alpine flowers and bumble bees: Its mechanism and impacts on the population dynamics of bumble bees.  G. Kudo et al. Oecologia (2025)

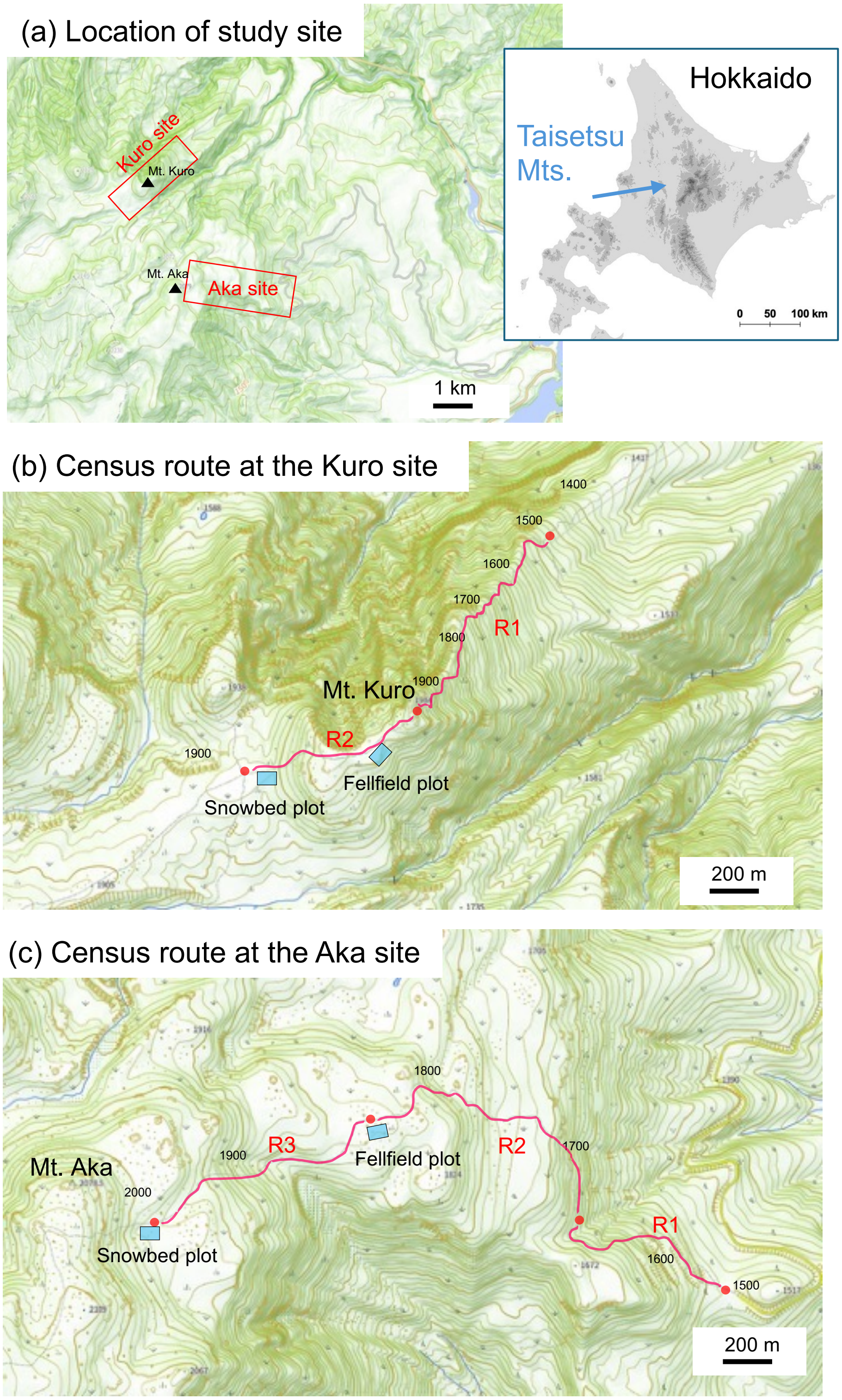


**Fig. S1.** (a) Location of study sites in the Taisetsu Mountain of Hokkaido. (b) and (c) The census route of bumble bees and the monitoring plots of flowering phenology in the fellfield and snowbed communities at the Kuro and Aka sites. Census routes were set along hiking trails (from 1500 m to 2000 m a.s.l.) and divided into two (Kuro site) or three (Aka site) parts.

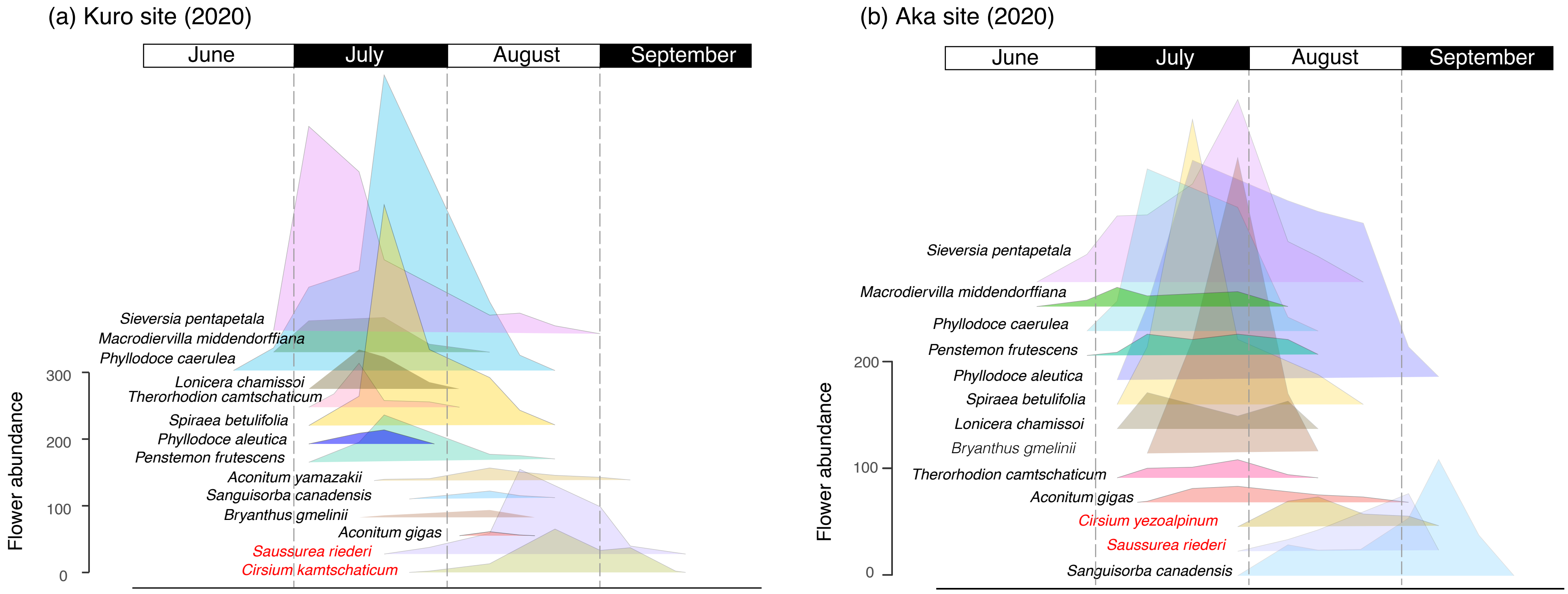


**Fig. S2.** Seasonal changes in flower abundance of the main plant species visited by bumble bees at the Kuro site (a) and the Aka site (b) in 2020. Flower or inflorescence abundance of each species was recorded at 7-10 days intervals from late June to early September in 40–50 quadrats (1 m x 1 m) placed along the trail to quantify the amount of flower resources for bumble bees at each site. Thistle species are shown in red. (G. Kudo and T. Nagase, unpublished data).

**Table S1.** Species list observed in the monitoring plots of flowering phenology. Plant group is classified as early-flowering fellfield plants (FE), late-flowering fellfield plants (FL), and snowbed plants (S). Plant species growing at both fellfield and snowbed habitats are separately listed in each category. Circles mean the occurrence of flowering in each year (2010-2023).

**(a) Kuro site**

| Species | Group | Y10 | Y11 | Y12 | Y13 | Y14 | Y15 | Y16 | Y17 | Y18 | Y19 | Y20 | Y21 | Y22 | Y23 |
|---|---|---|---|---|---|---|---|---|---|---|---|---|---|---|---|
| *Arctous alpina* var. *japonica* | FE | ○ | ○ | ○ | ○ | ○ | ○ | ○ | ○ | ○ | ○ | ○ | ○ | ○ | ○ |
| *Arcterica nana* | FE | ○ | ○ | ○ | ○ | ○ | ○ | ○ | ○ | ○ | ○ | ○ | ○ | ○ | ○ |
| *Loiseleuria procumbens* | FE | ○ | ○ | ○ | ○ | ○ | ○ | ○ | ○ | ○ | ○ | ○ | ○ | ○ | ○ |
| *Potentilla matsumurae* | FE | ○ | ○ | ○ | ○ | ○ | ○ | ○ | ○ | ○ | ○ | ○ | ○ | ○ | ○ |
| *Diapensia lapponica* var. *obovata* | FE | ○ | ○ | ○ | ○ | ○ | ○ | ○ | ○ | ○ | ○ | ○ | ○ | ○ | ○ |
| *Potentilla miyabei* | FE | ○ | ○ | ○ | ○ | ○ | ○ | ○ | ○ | ○ | ○ | ○ | ○ | ○ | ○ |
| *Vaccinium uliginosum* | FE | ○ | ○ | ○ | ○ | ○ | ○ |  | ○ | ○ | ○ | ○ | ○ | ○ | ○ |
| *Patrinia sibirica* | FL | ○ | ○ | ○ | ○ | ○ | ○ | ○ | ○ | ○ | ○ | ○ | ○ | ○ | ○ |
| *Spiraea betulifolia* var. *aemiliana* | FL | ○ | ○ | ○ | ○ | ○ | ○ | ○ | ○ | ○ | ○ | ○ | ○ | ○ | ○ |
| *Therorhodion camtschaticum* | FL | ○ | ○ | ○ | ○ | ○ | ○ | ○ | ○ | ○ | ○ | ○ | ○ | ○ | ○ |
| *Pennellianthus frutescens* | FL | ○ | ○ | ○ | ○ | ○ | ○ | ○ | ○ | ○ | ○ | ○ | ○ | ○ | ○ |
| *Cassiope lycopodioides* | FL |  |  | ○ | ○ |  |  | ○ |  |  |  | ○ | ○ |  | ○ |
| *Dicentra peregrina* | FL | ○ | ○ |  |  |  | ○ |  |  |  |  | ○ | ○ |  | ○ |
| *Bryanthus gmelinii* | FL | ○ | ○ | ○ | ○ | ○ | ○ | ○ | ○ | ○ | ○ | ○ | ○ | ○ | ○ |
| *Tilingia ajanensis* | FL | ○ | ○ | ○ | ○ | ○ | ○ | ○ | ○ | ○ | ○ | ○ | ○ | ○ | ○ |
| *Campanula lasiocarpa* | FL | ○ | ○ | ○ | ○ |  | ○ | ○ | ○ |  | ○ | ○ | ○ | ○ |  |
| *Saussurea yanagisawae* | FL | ○ | ○ |  | ○ |  | ○ |  | ○ |  |  | ○ |  |  | ○ |
| *Harrimanella stelleriana* | S | ○ | ○ | ○ | ○ | ○ | ○ | ○ | ○ | ○ | ○ | ○ | ○ | ○ | ○ |
| *Primula cuneifolia* | S | ○ | ○ | ○ | ○ | ○ | ○ | ○ | ○ | ○ | ○ | ○ | ○ | ○ | ○ |
| *Rhododendron aureum* | S | ○ | ○ | ○ | ○ | ○ | ○ | ○ | ○ | ○ | ○ | ○ | ○ | ○ | ○ |
| *Loiseleuria procumbens* | S | ○ | ○ | ○ | ○ | ○ | ○ | ○ | ○ | ○ | ○ | ○ | ○ | ○ | ○ |
| *Arcterica nana* | S |  |  |  | ○ |  |  |  |  |  |  |  |  |  |  |
| *Phyllodoce caerulea* var. *yezoensis* | S | ○ | ○ | ○ | ○ | ○ | ○ | ○ | ○ | ○ | ○ | ○ | ○ | ○ | ○ |
| *Sieversia pentapetala* | S | ○ | ○ | ○ | ○ | ○ | ○ | ○ | ○ | ○ | ○ | ○ | ○ | ○ | ○ |

| | | | | | | | | | | | | | | | |
|---|---|---|---|---|---|---|---|---|---|---|---|---|---|---|---|
| *Potentilla matsumurae* | S | ○ | ○ | ○ | | | ○ | | | | ○ | ○ | ○ | | |
| *Cassiope lycopodioides* | S | | ○ | ○ | ○ | | ○ | ○ | | | | | ○ | | |
| *Vaccinium vitis-idaea* | S | | | | | | ○ | | | | | | ○ | | |
| *Pediculasis chamissonis* | S | ○ | ○ | ○ | ○ | ○ | ○ | ○ | ○ | | ○ | ○ | | | |
| *Gentiana nipponica* | S | ○ | ○ | ○ | ○ | ○ | ○ | ○ | ○ | ○ | ○ | ○ | ○ | ○ | ○ |
| *Solidago virgaurea* subsp. *leiocarpa* | S | ○ | ○ | ○ | ○ | ○ | ○ | ○ | ○ | ○ | ○ | ○ | | ○ | ○ |
| *Tilingia ajanensis* | S | ○ | ○ | ○ | ○ | ○ | ○ | ○ | ○ | ○ | ○ | ○ | | | ○ |
| *Campanula lasiocarpa* | S | ○ | ○ | ○ | ○ | ○ | ○ | ○ | ○ | ○ | ○ | ○ | | ○ | ○ |

**(b) Aka site**

| Species | Group | Y10 | Y11 | Y12 | Y13 | Y14 | Y15 | Y16 | Y17 | Y18 | Y19 | Y20 | Y21 | Y22 | Y23 |
|---|---|---|---|---|---|---|---|---|---|---|---|---|---|---|---|
| *Arctous alpina* var. *japonica* | FE | ○ | ○ | ○ | ○ | ○ | ○ | ○ | ○ | ○ | ○ | ○ | ○ | ○ | ○ |
| *Loiseleuria procumbens* | FE | ○ | ○ | ○ | ○ | ○ | ○ | ○ | ○ | ○ | ○ | ○ | ○ | ○ | ○ |
| *Diapensia lapponica* var. *obovata* | FE | ○ | ○ | ○ | ○ | ○ | ○ | ○ | ○ | ○ | ○ | ○ | ○ | ○ | ○ |
| *Potentilla matsumurae* | FE | ○ | ○ | ○ | ○ | ○ | ○ | ○ | ○ | ○ | ○ | ○ | ○ | ○ | ○ |
| *Potentilla miyabei* | FE | ○ | ○ | ○ | ○ | ○ | ○ | ○ | ○ | ○ | ○ | ○ | ○ | ○ | ○ |
| *Vaccinium uliginosum* | FE | ○ | ○ | ○ | ○ | ○ | ○ | ○ | ○ | ○ | ○ | ○ | ○ | ○ | ○ |
| *Dicentra peregrina* | FE | ○ | ○ | ○ | ○ | ○ | ○ | ○ | ○ | ○ | ○ | ○ | ○ | ○ | ○ |
| *Rhododendron subarcticum* | FE | | | | ○ | | | | | | | | | | |
| *Patrinia sibirica* | FL | ○ | ○ | ○ | ○ | ○ | ○ | ○ | ○ | ○ | ○ | ○ | ○ | ○ | ○ |
| *Pedicularis oederi* var. *heteroglossa* | FL | ○ | ○ | ○ | ○ | ○ | ○ | ○ | ○ | ○ | ○ | ○ | | ○ | ○ |
| *Spiraea betulifolia* var. *aemiliana* | FL | ○ | ○ | ○ | ○ | ○ | ○ | ○ | ○ | | ○ | ○ | ○ | ○ | ○ |
| *Bryanthus gmelinii* | FL | ○ | ○ | ○ | ○ | ○ | ○ | ○ | ○ | ○ | ○ | ○ | ○ | ○ | ○ |
| *Bistorta vivipara* | FL | ○ | ○ | | ○ | ○ | | | | | | ○ | ○ | | ○ |
| *Tilingia ajanensis* | FL | ○ | ○ | ○ | ○ | ○ | ○ | ○ | ○ | ○ | ○ | ○ | ○ | ○ | ○ |
| *Saussurea yanagisawae* | FL | ○ | ○ | ○ | ○ | ○ | ○ | ○ | ○ | ○ | ○ | ○ | ○ | ○ | ○ |
| *Gentiana algida* | FL | ○ | ○ | ○ | ○ | ○ | ○ | ○ | ○ | ○ | ○ | | ○ | ○ | ○ |
| *Rhododendron aureum* | S | ○ | ○ | ○ | ○ | ○ | ○ | ○ | ○ | ○ | ○ | ○ | ○ | ○ | ○ |
| *Primula cuneifolia* | S | ○ | ○ | ○ | ○ | ○ | ○ | ○ | ○ | ○ | ○ | ○ | ○ | ○ | ○ |

| | | | | | | | | | | | | | | | |
|---|---|---|---|---|---|---|---|---|---|---|---|---|---|---|---|
| *Harrimanella stelleriana* | S | ○ | ○ | ○ | ○ | ○ | ○ | ○ | ○ | ○ | ○ | ○ | ○ | ○ | ○ |
| *Potentilla matsumurae* | S | ○ | ○ | ○ | ○ | ○ | ○ | ○ | ○ | ○ | ○ | ○ | ○ | ○ | ○ |
| *Phyllodoce caerulea* var. *yezoensis* | S | ○ | ○ | ○ | ○ | ○ | ○ | ○ | ○ | ○ | ○ | ○ | ○ | ○ | ○ |
| *Sieversia pentapetala* | S | ○ | ○ | ○ | ○ | ○ | ○ | ○ | ○ | ○ | ○ | ○ | ○ | ○ | ○ |
| *Phyllodoce aleutica* | S | ○ | ○ | ○ | ○ | ○ | ○ | ○ | ○ | ○ | ○ | ○ | ○ | ○ | ○ |
| *Vaccinium uliginosum* | S | | | | | | | | | | | | | | ○ |
| *Gentiana nipponica* | S | ○ | ○ | ○ | ○ | ○ | ○ | ○ | ○ | ○ | ○ | ○ | ○ | ○ | ○ |
| *Veronica stelleri* var. *longistyla* | S | ○ | ○ | ○ | ○ | ○ | ○ | ○ | ○ | ○ | ○ | ○ | ○ | ○ | ○ |
| *Pediculasis chamissonis* | S | ○ | ○ | ○ | ○ | ○ | ○ | ○ | ○ | ○ | ○ | ○ | ○ | ○ | ○ |
| *Loiseleuria procumbens* | S | | | | | | | | | | | | | | ○ |
| *Peucedanum multivittatum* | S | ○ | ○ | ○ | ○ | ○ | ○ | ○ | ○ | ○ | ○ | ○ | ○ | ○ | ○ |
| *Solidago virgaurea* subsp. *leiocarpa* | S | ○ | ○ | ○ | ○ | ○ | ○ | ○ | ○ | ○ | ○ | ○ | ○ | ○ | ○ |
| *Anemone narcissiflora* var. *sachalinensis* | S | | | | | | | | | ○ | ○ | ○ | | | |
| *Sanguisorba stipulata* | S | | ○ | ○ | ○ | ○ | ○ | ○ | ○ | ○ | ○ | ○ | ○ | ○ | ○ |
| *Parnassia palustris* | S | | | | | ○ | | | | ○ | | | | ○ | ○ |
| *Cirsium kamtschaticum* ssp. *alpinum* | S | ○ | ○ | ○ | ○ | ○ | ○ | ○ | ○ | ○ | ○ | ○ | ○ | ○ | ○ |
| *Saxifraga merkii* | S | | ○ | ○ | | | ○ | | | ○ | ○ | ○ | ○ | ○ | |

**Table S2.** The results of linear models conducted for the peak time (day of year) of worker bees of four *Bombus* species. Explanatory variables in the full models were deviations in snowmelt time, early summer (June) and mid-summer (July–August) temperatures, and site (Kuro, Aka). Best-fit models based on AICc values are shown. AICc value of full model is shown in parenthesis.

| | Coeff. | SE | *t* value | *P* value |
|---|---|---|---|---|
| **(a)** ***B. hypocrita*** AICc = 149.9 (160.2) | | | | |
| Intercept | 100.7 | 1.04 | 96.7 | < 0.0001 *** |
| **(b)** ***B. beaticola*** AICc = 154.5 (172.6) | | | | |
| Intercept | 104.4 | 1.15 | 91.1 | < 0.0001 *** |
| **(c)** ***B. yezoensis*** AICc = 148.8 (164.7) | | | | |
| Intercept | 107.3 | 1.00 | 106.9 | < 0.0001 *** |
| ΔSnowmelt time | 0.25 | 0.12 | 2.02 | 0.056 $^{+}$ |
| **(d)** ***B. hypnorum*** AICc = 144.5 (164.2) | | | | |
| Intercept | 93.5 | 1.50 | 62.2 | < 0.0001*** |

Full model: Peak DOY ~ (ΔSnowmelt time + ΔEarly summer temp + ΔMid-summer temp) × Site

**Table S3.** The results of linear models conducted for phenological mismatch (day), i.e., difference between peak flowering time of snowbed plants and peak time of worker bees of four *Bombus* species. Explanatory variables in the full models were deviations in snowmelt time, early summer (June) and mid-summer (July–August) temperatures, and site (Kuro, Aka). Best-fit models based on AICc values are shown. AICc value of full model is shown in parenthesis.

| | Coeff. | SE | *t* value | *P* value |
|---|---|---|---|---|
| **(a)** ***B. hypocrita*** AICc = 166.1 (183.2) | | | | |
| Intercept (Kuro site) | 6.40 | 1.94 | 3.30 | 0.0034 |
| ΔSnowmelt time | -0.68 | 0.17 | -4.06 | 0.0006 *** |
| Aka site | -8.41 | 2.72 | -3.09 | 0.0055 ** |
| **(b)** ***B. beaticola*** AICc = 164.2 (191.7) | | | | |
| Intercept (Kuro site) | 12.78 | 1.87 | 6.84 | < 0.0001 *** |
| ΔSnowmelt time | -0.71 | 0.16 | -4.36 | 0.0003 *** |
| Aka site | -13.83 | 2.62 | -5.28 | < 0.0001 *** |
| **(c)** ***B. yezoensis*** AICc = 148.4 (165.4) | | | | |
| Intercept (Kuro site) | 13.92 | 1.35 | 10.34 | < 0.0001 *** |
| ΔSnowmelt time | -0.52 | 0.12 | -4.43 | 0.0002 *** |
| Aka site | -10.56 | 1.89 | -5.60 | < 0.0001 *** |
| **(d)** ***B. hypnorum*** AICc = 137.6 (163.3) | | | | |
| Intercept (Kuro site) | 0.10 | 2.26 | 0.05 | 0.97 |
| ΔSnowmelt time | -0.92 | 0.20 | -4.54 | 0.0003 *** |
| Aka site | -12.99 | 3.41 | -3.81 | 0.0015 ** |

Full model: mismatch ~ (ΔSnowmelt time + ΔEarly summer temp + ΔMid-summer temp) × Site
